# Kikuchi Disease in a Young Woman With Sickle Cell Disease: A Case Report

**DOI:** 10.7759/cureus.78950

**Published:** 2025-02-13

**Authors:** Jumanah Alfuwayris, Mashael Almousa, Abdulaziz Alsarawi

**Affiliations:** 1 Rheumatology, King Abdulaziz Hospital, Al Ahsa, SAU; 2 Internal Medicine, King Abdulaziz Hospital, Al Ahsa, SAU

**Keywords:** fever, kikuchi fujimoto disease, lymphadenopathy, necrotizing lymphadenitis, rare diseases, sickle cell disease

## Abstract

Kikuchi-Fujimoto disease (KFD) is a rare, benign, and self-limiting disorder characterized by necrotizing lymphadenitis, primarily affecting young adults and adolescents. Its etiology and pathogenesis remain unclear and complex. Common presentations include constitutional symptoms such as lymphadenopathy and fever.

This study reports the case of a 15-year-old girl who presented with a fever of unknown origin and was found to have lymphadenopathy, which was associated with sickle cell disease (SCD). The diagnosis was confirmed through the pathological features of lymph node biopsy, with the patient successfully treated with steroids and hydroxychloroquine. She remained stable on follow-up, with no recurrence of fever or lymphadenopathy.

## Introduction

Kikuchi-Fujimoto disease (KFD), also known as histiocytic necrotizing lymphadenitis, is a rare, self-limiting condition that presents significant diagnostic challenges [1-3]. Initially reported in 1972 in Japan [1], KFD typically presents with nonspecific and constitutional symptoms, including fever and unilateral lymphadenopathy, predominantly affecting cervical lymph nodes [2,4]. The disease is more prevalent in females, with a reported female-to-male ratio of 4:1 [2-4]. While KFD is generally self-limiting, some patients may require immunosuppressive treatments such as corticosteroids [5,6].

We present the case of a young woman with sickle cell disease (SCD) who developed fever and lymphadenopathy. After a comprehensive histopathological analysis of her lymph nodes, she was diagnosed with KFD. This case highlights the need for awareness of KFD in patients with underlying hematological disorders, as it can mimic other conditions and complicate clinical management.

## Case presentation

A 15-year-old girl with a history of SCD presented to the emergency department with fever and vasoocclusive pain. On admission, her vital signs were as follows: blood pressure 107/58 mmHg, heart rate 113 beats per minute, respiratory rate 19 breaths per minute, temperature 39.2°C, and oxygen saturation 98% on room air. Physical examination was unremarkable. The patient’s laboratory results revealed a white blood cell count of 7.66 × 10⁹/L, hemoglobin of 89 g/L, platelet count of 420 × 10⁹/L, creatinine of 44 µmol/L, C-reactive protein of 23.9 mg/L, alanine aminotransferase of 40 U/L, and aspartate aminotransferase of 68 U/L. A comprehensive septic evaluation and cultures yielded negative results. She received empiric antibiotic therapy, along with intravenous fluids and pain management. Despite these interventions, the fever persisted, prompting further investigation.

**Figure 1:**
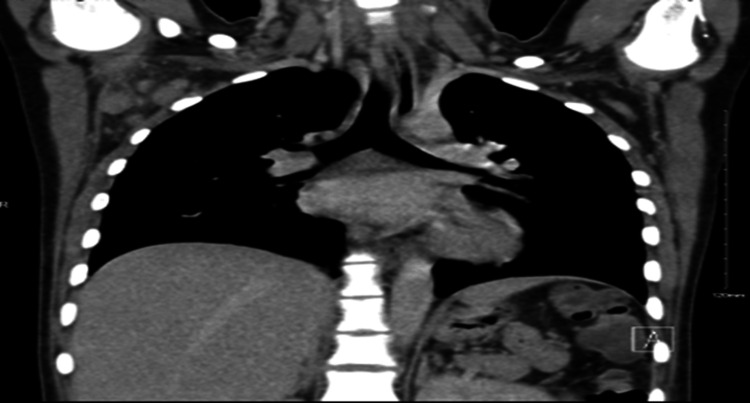
Whole body CT scan identified right axillary lymphadenopathy

**Figure 2:**
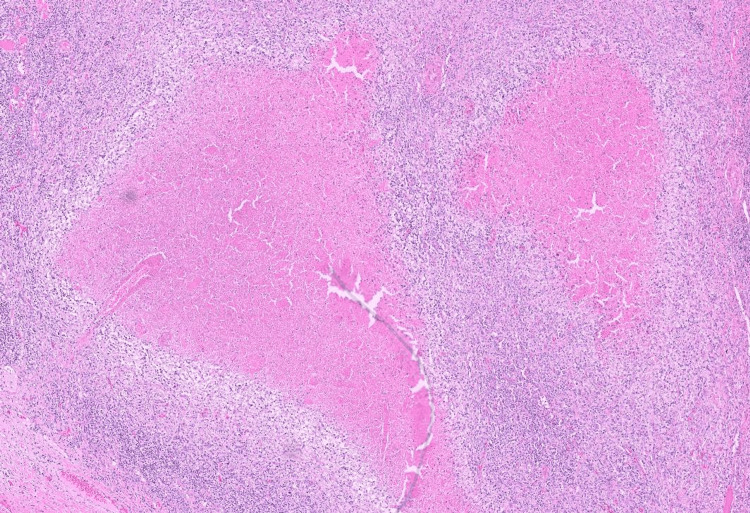
Axillary lymph node pathology 1

**Figure 3:**
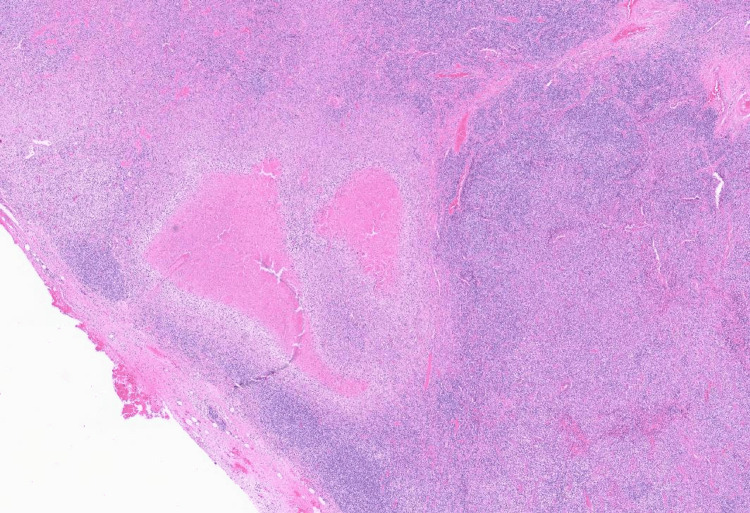
Axillary lymph node pathology 2

Imaging studies, including a white blood cell scan, revealed no focal infectious sites; however, a whole-body computed tomography (CT) scan identified right axillary lymphadenopathy (Figure 1). Histopathological and microscopic examination of the lymph node revealed necrotic areas surrounded by xanthomatous cells, along with eosinophilic granular material, karyorrhectic debris, interspersed lymphocytes, and histiocytes, consistent with KFD (Figures 2, 3). The autoimmune workup revealed no significant findings, including negative results for anti-nuclear antibody, anti-double stranded DNA antibody, and anti-Smith antibody. Given the presentation, KFD could mimic systemic lupus erythematosus (SLE) or potentially coexist with it. The patient was discharged on prednisolone, and hydroxychloroquine was subsequently added to her treatment regimen to manage recurrent fever. After the regimen modification, she has remained stable and is being followed up in the outpatient clinic. A follow-up visit after one month revealed an improved clinical condition with no further fever attacks.

## Discussion

Kikuchi-Fujimoto disease is a rare but benign disorder reported across all racial and ethnic groups worldwide, with a significantly higher prevalence in Asian individuals. It primarily affects young adults of both sexes although its frequency can vary, with reports indicating a higher prevalence in females [1,7].

The underlying etiology of KDF remains unclear; however, some cases have been associated with autoimmune diseases, particularly SLE [8,9], which can be challenging to differentiate from KFD owing to similar clinical and pathological features [10].

Our patient was diagnosed with KFD based on clinical presentation and histopathological examination findings of histiocytic necrotizing lymphadenitis, which is considered the gold standard for diagnosis [8,10].

Owing to its rarity, the treatment of KFD has not been extensively studied. However, several reported cases have demonstrated outstanding responses to various types of treatment such as nonsteroidal anti-inflammatory drugs (NSAIDs), corticosteroids, hydroxychloroquine, intravenous immunoglobulin, and anakinra, particularly in cases with recurrent or severe manifestations [1,3].

The association between SCD and KFD has not been well-documented, with only a few reported cases. Several of these cases present with atypical clinical manifestations [11]. The reported cases suggest that ethnic and racial factors may play a significant role, given the high prevalence of SCD and SLE among individuals of African descent [3].

## Conclusions

Overall, this case report describes Kikuchi-Fujimoto disease (KFD) in a patient with SCD, who was successfully treated with a combination of prednisolone and hydroxychloroquine. Given the rarity of this concurrent presentation, healthcare providers should be vigilant in recognizing KFD to prevent misdiagnosis and ensure timely, appropriate treatment. Although KFD is typically a self-limiting disorder, its diagnosis can be challenging, particularly in patients with pre-existing hematological conditions such as SCD. The co-occurrence of these two conditions raises important clinical questions and underscores the need for further investigation into the potential relationship between SCD and KFD.

This case highlights the significance of including KFD in the differential diagnosis of lymphadenopathy in patients with underlying hematological disorders, particularly SCD. Further studies are essential to explore the pathophysiological mechanisms underlying the association between KFD and hematological disorders like SCD, which could ultimately contribute to more targeted and effective management strategies.
